# PathwayMultiomics: An R Package for Efficient Integrative Analysis of Multi-Omics Datasets With Matched or Un-matched Samples

**DOI:** 10.3389/fgene.2021.783713

**Published:** 2021-12-22

**Authors:** Gabriel J. Odom, Antonio Colaprico, Tiago C. Silva, X. Steven Chen, Lily Wang

**Affiliations:** ^1^ Department of Biostatistics, Stempel College of Public Health, Florida International University, Miami, FL, United States; ^2^ Department of Public Health Sciences, Miller School of Medicine, University of Miami, Miami, FL, United States; ^3^ Sylvester Comprehensive Cancer Center, Miller School of Medicine, University of Miami, Miami, FL, United States; ^4^ Dr. John T Macdonald Foundation Department of Human Genetics, Miller School of Medicine, University of Miami, Miami, FL, United States; ^5^ John P. Hussman Institute for Human Genomics, Miller School of Medicine, University of Miami, Miami, FL, United States

**Keywords:** pathway analysis, gene set analysis, multi-omics, integrative analysis, R package, Alzheheimer’s disease

## Abstract

Recent advances in technology have made multi-omics datasets increasingly available to researchers. To leverage the wealth of information in multi-omics data, a number of integrative analysis strategies have been proposed recently. However, effectively extracting biological insights from these large, complex datasets remains challenging. In particular, matched samples with multiple types of omics data measured on each sample are often required for multi-omics analysis tools, which can significantly reduce the sample size. Another challenge is that analysis techniques such as dimension reductions, which extract association signals in high dimensional datasets by estimating a few variables that explain most of the variations in the samples, are typically applied to whole-genome data, which can be computationally demanding. Here we present pathwayMultiomics, a pathway-based approach for integrative analysis of multi-omics data with categorical, continuous, or survival outcome variables. The input of pathwayMultiomics is pathway *p-*values for individual omics data types, which are then integrated using a novel statistic, the MiniMax statistic, to prioritize pathways dysregulated in multiple types of omics datasets. Importantly, pathwayMultiomics is computationally efficient and does not require matched samples in multi-omics data. We performed a comprehensive simulation study to show that pathwayMultiomics significantly outperformed currently available multi-omics tools with improved power and well-controlled false-positive rates. In addition, we also analyzed real multi-omics datasets to show that pathwayMultiomics was able to recover known biology by nominating biologically meaningful pathways in complex diseases such as Alzheimer’s disease.

## Introduction

Recent advances in technology have made multi-omics datasets increasingly available to researchers. For example, The Cancer Genome Atlas (TCGA) and the Clinical Proteomic Tumor Analysis Consortium (CPTAC) have generated comprehensive molecular profiles including genomic, epigenomic, and proteomic expressions on matched samples for many types of human tumors. The underlying hypothesis is that multiple types of molecular profiles (e.g., copy number, DNA methylation, protein) might provide a more coherent and complete signature of the disease process.

To leverage the wealth of information in multi-omics data, a number of integrative analysis strategies have been proposed (Meng et al., 2016; Huang et al., 2017) and compared (Le Cao et al., 2009; Pucher et al., 2019). These methods can be roughly classified into three different categories, characterized by the way they leverage information from the multi-omics datasets. The first group of methods (Parkhomenko et al., 2009; Waaijenborg and Zwinderman, 2009; Witten and Tibshirani, 2009; Lin et al., 2013) analyzes only intersecting (i.e., matched) samples from the multiple omics datasets and only shared genes measured by all types of omics platforms. The second group of methods (Dray and Dufour, 2007; Kaspi and Ziemann, 2020) analyzes only genes shared by multiple types of omics datasets, which may be measured on the same or distinct samples in different omics datasets. The third group of methods (Gao et al., 2004; Kutalik et al., 2008; Zhang et al., 2012; Meng et al., 2014) analyzes matched samples in multi-omics datasets, where each dataset may have the same or distinct genes.

Because of the complexities in multi-omics datasets, effectively extracting biological insights from these datasets remains challenging. A major challenge for multi-omics data analysis is that the samples are often measured on one or a few, but not all, omics data types. Therefore, multi-omics analysis tools that require matched samples (with measurements for all omics data types) as input can significantly limit the sample size when several omics data types are considered. Another challenge is that analysis techniques such as dimension reduction techniques are typically applied to genome-wide data, which can be computationally demanding. Thus, to maximally leverage information from the multi-omics datasets, there is a critical need for developing additional integrative methods that are not restricted to only matched samples and/or shared genes in the input datasets.

Here we present pathwayMultiomics, a pathway-based approach for integrative analysis of multi-omics data. Instead of testing individual genes, pathway analysis tests joint effects of multiple genes belonging to the same biological pathway, such as those defined in the KEGG (Kanehisa et al., 2012) database. Higher power in the pathway-based analysis is achieved by combining weak signals from a number of individual genes in the pathway (Subramanian et al., 2005). The input of pathwayMultiomics is pathway *p-*values for individual omics data types, which are then integrated using a novel statistic, the MiniMax statistic, to prioritize pathways dysregulated in multiple types of omics datasets. Because pathwayMultiomics only requires summary statistics (i.e., pathway *p-*values) as input, it is computationally efficient. In addition, it is also flexible and can be used to analyze multi-omics datasets with categorical, continuous, or survival outcome variables. Importantly, using summary statistics as input allows pathwayMultiomics to maximally leverage information in multi-omics datasets by not restricting to only shared samples and/or genes. Using simulated datasets, we showed that pathwayMultiomics significantly outperforms currently available multi-omics methods with improved power and well-controlled false-positive rates. In addition, we also analyzed multi-omics datasets in Alzheimer’s disease to show that pathwayMultiomics was able to recover known biology by nominating biologically meaningful pathways.

## Materials and Methods

### An Overview of pathwayMultiomics Algorithm

**FIGURE 1:**
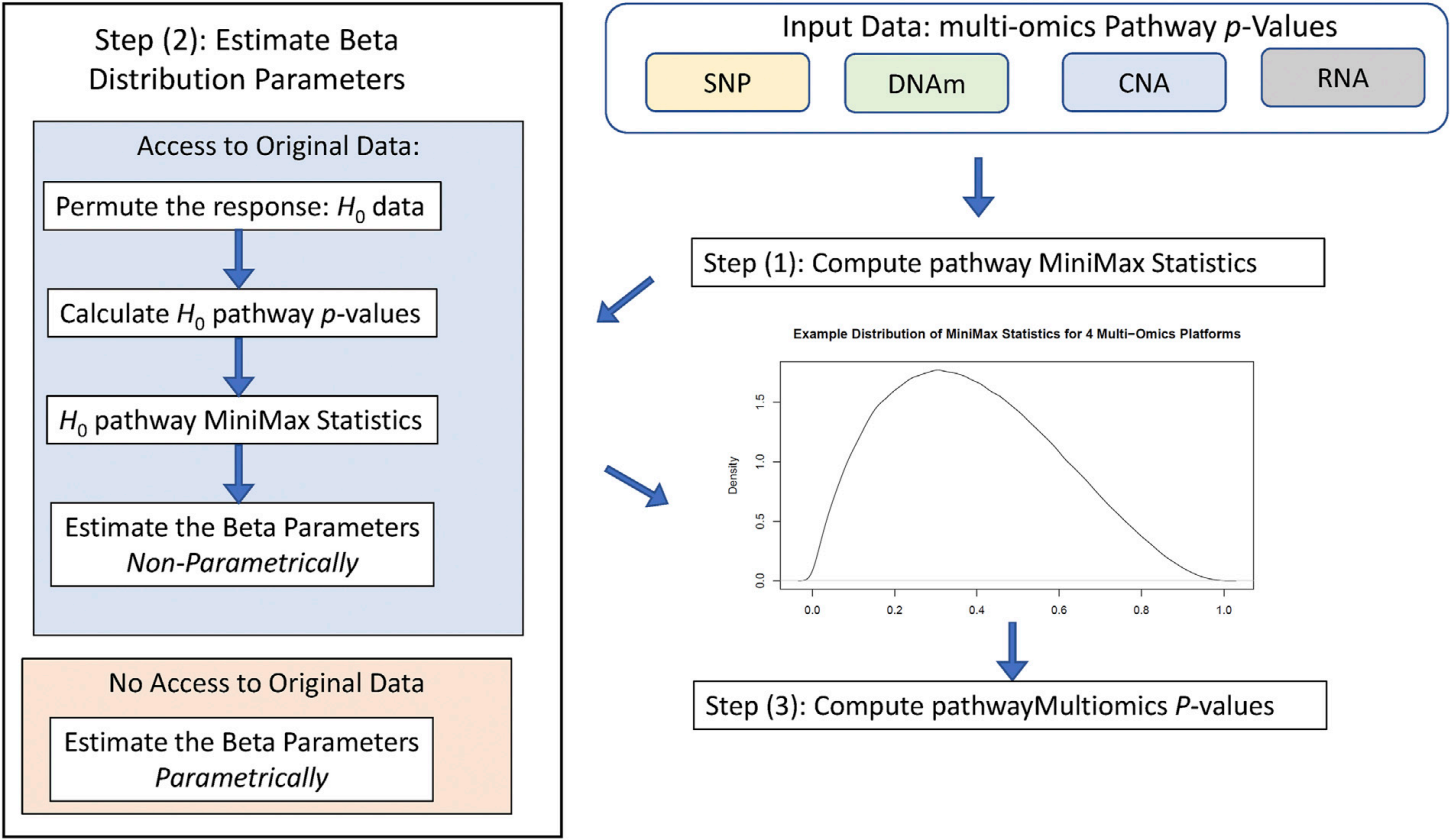
Workflow of pathwayMultiomics analysis.

Figure 1 illustrates the workflow of the pathwayMultiomics analysis pipeline. We next describe the input datasets, analytical algorithm, and output in detail. The pathwayMultiomics package for R can be accessed from https://github.com/TransBioInfoLab/pathwayMultiomics.

#### Input Datasets

The input dataset consists of omics datasets for several different molecular traits, such as SNPs, DNA methylation (DNAm), copy number alterations (CNAs), or gene expressions. Of particular interest are dysregulated pathways at multiple molecular levels, for example, those with changes in both DNA methylation and gene expressions. Importantly, pathwayMultiomics is flexible; the samples can be either matched (multiple types of molecular traits are measured on the same set of samples), or un-matched (distinct samples from the same disease are measured with different types of omics technology). Moreover, because the units of analyses for pathwayMultiomics are pathways (i.e., groups of genes participating in the same biological processes), different omics datasets can also include different genes, as long as pathway-level association statistics that relate each type of omics profiles to the phenotype (e.g., pathway *p-*values) can be computed. This flexibility enables pathwayMultiomics to take advantage of different pathway analysis software to model and account for special characteristics in different types of omics datasets. For example, for pathway analysis of DNAm data, the missMethyl method (Phipson et al., 2016), which takes account of the varying number of probes mapped to each gene, could be used. For pathway analysis of gene expression data, pathwayPCA method (Odom et al., 2020), which selects the coherent subset of genes before estimating and testing principal components with phenotypes, could be applied.

#### MiniMax Statistic

Given pathway *p-*values for each omics data type, pathwayMultiomics next computes the MiniMax statistic. To this end, we first consider all pairs of *p-*values from different omics types and take the maximum for each pair of *p-*values. Next, we take the **mini**mum of all **max**imums computed from the last step. For example, suppose we are interested in an apoptosis pathway for a cancer study, which has *p-*values of 0.01, 0.03, and 0.05 for copy number variations, gene expressions, and protein assays, respectively. We then have a total of three pairs of *p-*values (0.01, 0.03), (0.01, 0.05), (0.03, 0.05), with maximums 0.03, 0.05, and 0.05 respectively. The MiniMax statistic is the smallest value of these maximums, which is 0.03. Intuitively, the MiniMax statistic provides a way to identify pathways with differential changes (i.e., small *p-*values) in *at least two* types of omics data. Note that in this case, the MiniMax statistic is equivalent to taking the second smallest *p-*value among all *p-*values; that is, the second-order statistic, *P*_(2)_, of the pathway *p-*values. Instead of considering pairs of *p-*values, the MiniMax statistic can also be computed for triplets or quadruplets of *p-*values from three, four, or more types of omics data similarly to identify pathways with differential changes (i.e., small *p*-values) in more than two types of omics data.

#### Statistical Significance Assessment

To compute *p-*values for the MiniMax statistic, pathwayMultiomics has two modes: 1) by approximation or 2) by simulation. More specifically, the “approximation” approach is based on the theory that when different types of omics data are independent, the *r*th order statistic *p*_(r)_ of the *p*-values follows a Beta distribution, that is, $P_{\left(r\right)}\;∼\;ℬ\left(\alpha =r,\beta =G-r+1\right)$ , where ℬ(⋅,⋅) denotes the Beta distribution and G is the number of different types of omics data (Gentle, 2009; Jones, 2009). Therefore, for integrative analysis that identifies pathways with differential changes in at least two types of omics datasets, the MiniMax statistic is the second-order statistic and has the distribution $P_{\left(2\right)}\;∼\;ℬ\left(2,\;3-2+1\right)=ℬ\left(2,2\right)$ under the null hypotheses. The “approximation” approach is easy to compute and is useful when computational resources are limited or when raw data in different omics data types are not available.

On the other hand, in the “simulation” approach, we simulate the distribution of MiniMax statistics under the null hypothesis, that is, when there is no association between phenotype and the pathway in each type of omics data. More specifically, we generate random phenotype labels for each sample and then re-compute pathway *p-*values. These resulting *p-*values are our empirical null *p-*values. To account for non-independence in the different data types, instead of using the above formula, we estimate values for α and β from the empirical null *p-*values. In practice, we have found that the more correlated the *p*-values are across the multi-omics platforms, the smaller $\left(\hat{\alpha }<2,\hat{\beta }<G-1\right)$ are. The “simulation” approach provides more accurate statistical significance estimation and is recommended when both raw data for different omics and large computational resources are available.

#### Output

The output of pathwayMultiomics is prioritized pathways with small *p-*values in multiple omics data types, the MiniMax statistic and significance level for each pathway, and the omics data types that were contributing to the MiniMax statistic. For example, in the apoptosis pathway example we described above, the MiniMax statistic was 0.03, its *p*-value (using the approximate ℬ(2,2) distribution) would be 0.0026, and the omics data that contributed to MiniMax statistic were the copy number variations and gene expression data.

### Design of Simulation Studies

We performed a comprehensive simulation study to evaluate and compare the performance of the proposed pathwayMultiomics approach with four alternative methods for prioritizing pathways enriched with concordant but often subtle associations signals. To simulate multi-omics datasets with realistic correlation patterns, we used the TCGA COADREAD dataset (Vasaikar et al., 2018) as our input dataset, which included 614, 222, and 90 samples of copy number alterations (CNAs), gene expression, and proteomics data, respectively. More specifically, the CNA data included gene-level GISTIC2 log_2_ ratios for 24,776 genes; gene expression data included normalized counts ( $\log_{2}\left(x+1\right)$ transformation) of 6,149 genes generate by the Illumina GenomeAnalyzer platform; and the proteins data include log-ratio normalized protein expression levels of 5,538 genes.

To simulate multi-omics datasets for a collection of pathways, we first created synthetic pathways by performing hierarchical clustering on the 1,710 genes measured by all three types of assays for CNA, gene expression, and protein. More specifically, first, a data matrix with 1,710 genes and 928 samples (from the 623 subjects with at least one type of omics data) was created. Next, within each data type, data for each gene were centered and scaled. Finally, a modified Ward’s method (method = “ward.D” in hclust() function) was then used to partition the genes into 50 clusters or 50 synthetic pathways. The number of genes in the resulting pathways ranged from 9 to 74, with an average of 34 genes.

Next, we simulated treated (i.e., true positive) and un-treated (i.e., true negative) pathways. First, we randomly assigned each of the 623 subjects to one of two cancer subtypes: A or B. Next, among the 50 synthetic pathways, we selected five pathways to be our true positive pathways, and treatment effects at different levels (µ = 0.1, 0.2, 0.3, 0.4, 0.5) were added to a subset of genes (*p* = 20, 40, 60, 80%) within each pathway in each of the multi-omics datasets for samples in subtype A group. This process was then repeated 100 times to create 100 simulated multi-omics datasets, each including 50 pathways, among which 5 pathways are true positive pathways. Overall, we generated datasets for a total of 20 simulation scenarios (5 values for µ × 4 values for *p*). This benchmark dataset (available at https://zenodo.org/record/5683002#.YZF5SGDMKUk), which was systematically modified from real multi-omics data, can be used for reproducing analyses in this study as well as benchmarking future multi-omics data analysis methods.

To evaluate the false positive rate of each method, we also repeated the same procedures described above, except by setting µ = 0 (i.e., not adding any treatment effect). Multi-omics data was created for a total of 5,000 pathways by generating random sample labels 100 times for the 50 synthetic pathways. The false-positive rate (i.e., test size) for each method was then estimated by the percentage of pathways *p*-values less than 0.05.

Given the known status of the pathways, we next computed the area under the ROC curve (AUC) for each method. The receiver operating characteristic (ROC) curves is a plot of sensitivity versus 1-specificity as the cutoff for declaring significant pathways is varied. AUC assesses the overall discriminative ability of the methods to determine whether a given pathway is significantly associated with the phenotype (i.e., subtype group of the samples) over all possible significance cutoffs. More specifically, for each of the simulation scenarios, we recorded the rankings of the 50 pathways from most to least extreme (by either a *p*-value, test statistic, or score returned by a method), constructed ROC curves, and estimated AUC for each method.

### Methods Compared in the Simulation Study

We compared pathwayMultiomics with four alternative multi-omics analysis methods: Sparse Multiple Canonical Correlation Analysis (sparse mCCA) (Witten and Tibshirani, 2009), MFA (Dray and Dufour, 2007), iProFun (Song et al., 2019), and mitch (Kaspi and Ziemann, 2020). We chose mCCA to represent multi-omics matrix factorization techniques because it performed best in a recent comparative study of multi-omics analysis methods (Pucher et al., 2019). The last three methods, mitch, iProFun, and MFA were chosen because they were proposed in recent years and can also be applied to un-matched or partially matched datasets (Table 1). Note that each of these tools was designed specifically for the analysis of multi-omics data, either matching by samples, genomic features (e.g., gene or probe), or both. In the following, we briefly describe each of the methods compared in our simulation study. In the following, we briefly describe each of the methods compared in our simulation study.

**TABLE 1:** Methods compared by simulation study. Methods that analyze only matched samples would require multiple types of molecular data (e.g., gene expression and protein) to be generated for the same subject, methods that analyzes only matched genes would require multiple types of molecular data to be generated for the same gene. Summary data refers to resulting statistics such as *p*-values or *t*-statistics from differential expression analysis for genes or pathways. All function calls used default function arguments unless specified.

Method	Matches on	Analyzes only matched samples	Analyzes only matched genes	Can analyze summary data	Implementation R package::function
sCCA	Samples measured by all omics data types	Yes	Yes	No	PMA::MultiCCA.permute() with nperms = 100; and PMA::MultiCCA()
MFA	Features (e.g., genes)	No	Yes	No	ade4::ktab.list.df() and ade4:mfa() with option = “lambda1”
mitch	Features (e.g., genes)	No	Yes	Yes	mitch::mitch_calc() with minsetsize = 5 and priority = “effect”
iProFun	Samples measured on at least two omics data types	No	Yes	No	iProFun::iProFun_permutate() with parameters in package example (pi = rep (0.05, 2); grids = c (seq (0.75, 0.99, 0.01), seq (0.991, 0.999, 0.001), seq (0.9991, 0.9999, 0.0001)); filter = 1; seed = 123).
pathwayMultiomics	Pathways	No	No	Yes	pathwayMultiomics:MiniMax() with parameters orderStat = 2 and method = “parametric"

Abbreviations: sCCA, Sparse Canonical Correlates Analysis; MFA, Multi-Factor Analysis; mitch, multivariate gene set enrichment analysis; iProFun, Integrative Proteogenomic Functional Traits Analysis.

#### pathwayMultiomics

To compute pathway *p-*values for single omics data, we used pathwayPCA R package (Odom et al., 2020). PathwayPCA integrates prior biological knowledge to extract Adaptive Elastic-net Sparse PCs (AES-PCs) within each pathway for each omics dataset separately, the first AES-PC with the largest variance was then tested against binary outcome “cancer subtype” using a logistic regression model. The pathway *p-*values for each type of omics data were then used as input for pathwayMultiomics, to identify pathways dysregulated in more than one omics data type. Because the pathway *p-*values are calculated for each omics dataset separately, the statistical accuracy and power in pathwayMultiomics analysis will not change as the number of matched samples or shared features decreases.

#### Sparse Multiple Canonical Correlates Analysis (sCCA)

Sparse Canonical Correlation Analysis (sCCA) is a matrix factorization method that uses penalized multivariate analysis for identifying linear combinations of two groups of variables that are highly correlated. Witten and Tibshirani (2009) (Witten and Tibshirani, 2009) extended sCCA to sparse multiple CCA (mCCA), which can perform integrative analysis of more than two sets of variables measured on the same subjects. In the first step, sparse mCCA finds the set of intersecting (i.e., shared) samples and genes across all multi-omics datasets, i.e., the same set of genes are measured on the same subjects in each of the omics datasets. Therefore, the statistical accuracy and power of sparse mCCA to detect multi-omics changes will decrease as the number of shared samples or features decreases because samples or features not shared across all data sets will be discarded. In particular, in the TCGA COADREAD multi-omics datasets, only 71 samples and 1710 genes were measured on all three omics data types (CNA, gene expression, protein). Next, sparse mCCA uses a permutation procedure to determine the thresholds and to extract a single vector of selected genes for each omics data type. The union of these selected genes from each omics data type is then taken as the genes selected by sparse multiple CCA. Finally, a Fisher’s Exact Test is used to determine if a pathway is enriched with selected genes. We used mCCA implemented via the MultiCCA() function in the PMA R package (https://cran.r-project.org/web/packages/PMA/index.html), optimal weights and penalties were identified by the MultiCCA.permute() function.

#### Multi-Factor Analysis (MFA)

The MFA method is also a matrix factorization technique, but it differs from sparse mCCA in that it only requires data to be matched on features rather than samples. For MFA analysis of multi-omics data, the main requirement is that the same set of *p* genes are measured on all omics data types on potentially different subjects. Therefore, the statistical accuracy and power of MFA to detect multi-omics changes will not be affected by the number of matched samples, but will decrease as the number of shared features decreases, because features not shared across all data sets will be discarded. In the first step, MFA reshapes data by stacking the multi-omics datasets, each with samples as rows and the same *p* genes as columns. Next, MFA performs a weighted principal components analysis, where the weights from each data set are inversely related to the principal eigenvalue of the data set (a measurement of the overall variability in the dataset). Then, genes are given a score measuring its concordance across the datasets for different omics types, where the distribution of these scores follows *N* (*0, p* ^−1/2^) where *p* is the number of genes measured on all omics data types. Finally, genes with upper-sided *p*-values <0.05 are selected, and Fisher’s Exact Test is used to identify pathways significantly enriched with selected genes. We implemented the MFA method using the mfa() function in ade4 R package under default settings.

#### Multi-Contrast Pathway Enrichment Analysis (mitch)

**FIGURE 2:**
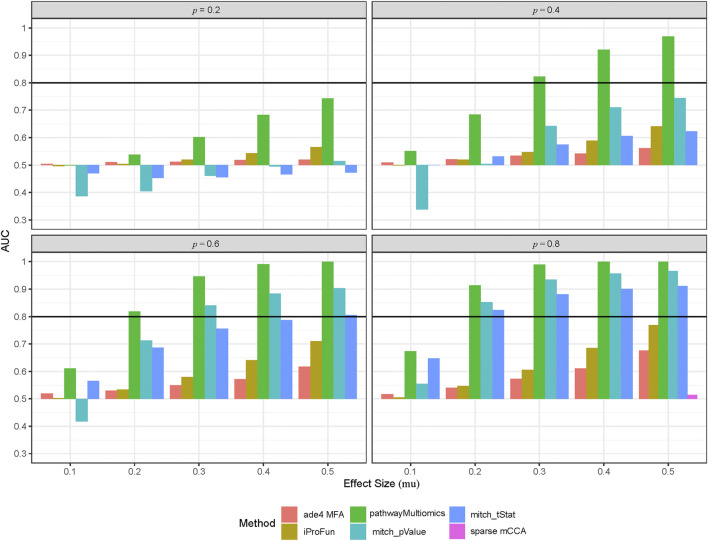
Performance of different multi-omics analysis methods in the simulation study. To simulate multi-omics datasets, we used the TCGA COADREAD datasets (in copy number alterations, gene expressions and proteomics data) as an input, created 50 synthetic pathways by clustering genes measured by all three types of omics data, and then added treatment with different effect sizes (mu) to a proportion (p = 0.2, 0.4, 0.6, 0.8) of the genes. This process was repeated for 100 times to create 100 simulated multi-omics datasets for each simulation scenario (i.e., different combinations of mu and p). Shown are area under ROC curves (AUCs) for each method averaged over 100 simulation datasets at each simulation scenario.

The mitch method is very similar to the proposed MiniMax statistic because it also computes pathway-level enrichment scores from summary statistics rather than using the data itself. There are several steps in the mitch algorithm: first, users identify the set of p genes measured by all *G* omics data types, and subsets the multi-omics datasets to include only these p genes. Next, for each omics dataset, methods appropriate for each platform (e.g., DESeq2 for RNASeq data) are used to compute gene-wise summary statistics or gene scores (e.g., *p-*values or *t*-statistics) that associate each gene with the phenotype. This step produces a *p* × *G* data matrix (i.e., *p* genes × *G* omics data types). Therefore, the statistical accuracy and power of mitch to detect multi-omics changes will not be affected by the number of matched samples, but will decrease as the number of shared features decreases, because features not shared across all data sets will be discarded. Finally, for each pathway, mitch performs a one-way MANOVA to test if gene scores across the *G* omics data types are different for genes within the pathways compared to background genes. We compared the mitch algorithm, computed using the mitch_calc() routine from the mitch R package with priority = “effect”, with two alternative gene-wise summary statistics: the gene-specific *t*-statistic obtained after fitting a linear model that associated each gene with subtype group effect (labeled as “mitch_tStat” in Figure 2), and the gene-specific *p-*values from the same linear models (labeled as “mitch_pValue”). Note that using the *t*-statistic accounted for different directions of associations among genes while using the *p*-value did not.

#### Integrative Screening for Proteogenomic Functional Traits (iProFun)

The iProFun method (Song et al., 2019) aims to detect DNA copy numbers (CNA) and methylation alterations (DNAm) with downstream functional consequences in mRNA expression levels, global protein abundances, or phosphoprotein abundances. In the first step, iProFun fits three linear models, each with a molecular trait (mRNA, global protein, or phosphoprotein) as the outcome, and CNA or DNAm as the predictor, along with additional covariate variables (e.g., age, sex). Next, multiple comparison correction is applied to *p-*values of the predictor (CNA or DNAm) in each of the three linear models, and genes with at least one significant predictor are selected. Finally, Fisher’s Exact Test is used to identify pathways enriched with selected genes. Notably, iProFun allows more flexibility in the input dataset and can take advantage of samples not completely measured on all omics types. Specifically, iProFun requires samples to be measured by at least one genomic (e.g., copy number, DNA methylation) trait and at least one transcriptomic (i.e., mRNA) or proteomic (e.g., global, phosphor protein) trait, but it does not require samples to be measured by more than one genomic trait or more than one transcriptomic/proteomic traits. In the simulation study, the number of shared samples analyzed by iProFun were 216 (copy number and RNAseq) and 88 (copy number and proteomics). The statistical accuracy and power of sparse iProFun to detect multi-omics changes will decrease as the number of these shared samples (between copy number and RNAseq, or between copy number and proteomics) decreases, because samples not shared by at least two data sets will be discarded. In our simulation study, we used the iProFun_permutate() function in the iProFun package to independently predict synthetic gene expressions and proteomics data from simulated copy number aberrations. Default parameter values, as shown in package examples, were used for all functions.

### Analysis of Multi-Omics Datasets in Alzheimer’s Disease

#### pathwayMultiomics Analysis

We next applied pathwayMultiomics to analyze a set of multi-omics datasets in Alzheimer’s disease. The input of pathwayMultiomics analysis is pathway *p-*values for single omics data. Therefore, we first performed pathway analysis for genetic variants, DNAm, and gene expressions using the mixed model approach (Wang et al., 2011), MissMethyl (Phipson et al., 2016), and fgsea (Korotkevich et al., 2021) methods, which were specifically designed for pathway analyses of these different omics data types.

More specifically, for the analysis of genetic variants, Kunkle et al. (2019) (Kunkle et al., 2019) described a recent large meta-analysis of more than 90,000 individuals to identify genetic variants associated with AD. We downloaded summary statistics for individual variants obtained in this study from https://www.niagads.org/igap-rv-summary-stats-kunkle-p-value-data (“Kunkle_et al._Stage1_results.txt”). Next, we performed GWAS pathway analysis using the mixed model approach (Wang et al., 2011), which tested the combined association signals from a group of variants in the same pathway against the null hypothesis that there is no overall association between SNPs in a pathway and the outcome (i.e., AD status). An empirical null distribution, estimated using the bacon R package (van Iterson et al., 2017), was used to estimate the statistical significance of the pathways.

For the analysis of DNA methylation data, we recently performed a meta-analysis of more than 1,000 prefrontal cortex brain samples (Zhang et al., 2020) to identify epigenetic changes associated with AD Braak stage, a standardized measure of neurofibrillary tangle burden determined at autopsy. Braak scores range from 0 to 6, corresponding to increased severity of the disease (Braak and Braak, 1995). Supplementary Tables 1, 2 in Zhang et al. (2020) included summary statistics for 3,751 differentially methylated individual CpGs and 119 differentially methylated regions (DMRs) that reached a 5% FDR significance threshold in our meta-analysis. The combined collections of the significant individual CpGs and CpGs located in the DMRs were then used as input for pathway analysis via the MissMethyl R package (Phipson et al., 2016), which performs over-representation analysis by determining if AD Braak-associated CpGs are significantly enriched in a pathway. In particular, MissMethyl models the multiple probes mapped to each gene on the methylation arrays using the Wallenius’ noncentral hypergeometric test.

For the analysis of RNASeq data, we analyzed 640 samples of RNAseq data measured on postmortem prefrontal cortex brain samples in the ROSMAP AD study. Normalized FPKM (Fragments Per Kilobase of transcript per Million mapped reads) gene expression values generated by the ROSMAP AD study were downloaded from the AMP-AD Knowledge Portal (Synapse ID: syn3388564). For each gene, we assessed the association between gene expression and Braak stage. More specifically, for each gene, we fitted the linear model log2 (normalized FPKM values +1) ∼ Braak stage + ageAtDeath + sex + markers for cell types. The last term, “markers for cell types,” included multiple covariate variables to adjust for the multiple types of cells in the brain samples. Specifically, we estimated expression levels of genes that are specific for the five main cell types present in the CNS: ENO2 for neurons, GFAP for astrocytes, CD68 for microglia, OLIG2 for oligodendrocytes, and CD34 for endothelial cells, and included these as variables in the above linear regression model, as was done in a previous large study of AD samples (De Jager et al., 2014). This linear model identifies genes for which gene expressions are associated with AD Braak stage linearly (Zhang et al., 2020). For pathway analysis, we ranked each gene by *p-*values for the Braak stage in the above linear model, which was then used as input for the Fast Gene Set Enrichment Analysis (fgsea) (Korotkevich et al., 2021) software. The fgsea software performs pathway analysis of genome-wide gene expression data by determining if genes within a pathway are enriched on top of the gene list (ranked by gene-wise differential gene expression *p-*values) compared to the rest of the genes.

The pairwise correlations of *p-*values in individual omics data types are very small, at *ρ* = 0.0045 (SNP pathway *p*-values vs. DNAm pathway *p*-values), −0.0263 (SNP pathway *p*-values vs. RNAseq pathway *p*-values), and 0.0432 (DNAm pathway *p*-values vs. RNAseq pathway *p-*values). In pathwayMultiomics, we used the approximation approach, supported by the relatively low pairwise correlations in pathway *p-*values of individual omics data types.

#### mitch Analysis

The input of mitch R package is summary statistics for genes such as *p-*values for different types of omics data. For the GWAS meta-analysis results described in (Kunkle et al., 2019), we assigned SNPs to a gene if they were located within 5 kb upstream of the first exon or downstream of the last exon (Wang et al., 2011). Next, we represented each gene by the smallest *p-*value if there are multiple SNPs associated with it. To remove selection bias due to different numbers of SNPs associated with each gene (i.e., the smallest *p-*value for a gene with many SNPs is likely to be smaller than the smallest *p-*value for a gene with only a few SNPs), we next fit a generalized additive model using the R package gam: $Y_{i}∼f\left(n.links_{i}\right)$ where *Y*_*i*_ is - log_10_ transformation of the smallest *p-*value for gene *i*, *n. links*_*i*_ is the number of SNPs associated with gene *i,* and *f* is a spline function. We assumed gamma distribution for *Y*_*i*_, as under the null hypothesis of no association, *Y*_*i*_ follows the chi-square distribution (a special case of gamma distribution). The spline model allows us to model linear and nonlinear associations between the number of SNPs mapped to a gene and the strength of significance for the gene as previously described (Zhang et al., 2021). The residuals from this model, which represented -log_10_ transformation of the *p-*values with gene size effects removed, were then estimated, and used as input for genetic data in mitch.

Similarly, for the analysis of DNA methylation data, we assigned CpGs to genes based on Illumina annotation, represented each gene by the CpG with the smallest *p-*value, and removed the bias due to gene size using the same spline model described above, except *n. links*_*i*_ is the number of CpGs associated with gene *i.* The residuals from the spline model were then used as input for DNAm data in mitch.

For the analysis of RNAseq data, we used the R package fgsea (Korotkevich et al., 2021). For each gene, we fit a linear model log2 (normalized FPKM values +1) ∼ Braak stage + ageAtDeath + sex + markers for cell types. As described above, the last term, “markers for cell types” included covariate variables (marker gene expressions of ENO2, *GFAP, CD68, OLIG2, CD34*) to adjust for the multiple types of cells in the brain samples. The -log10 transformation of the *p-*values for the Braak stage in the above model was then used as input for RNASeq data in mitch.

All analyses were performed using the R software (version 4.0) and SAS software (version 9.4). We used the venny tool (Oliveros, 2007-2015). To account for multiple comparisons, we computed the false discovery rate using the method of Benjamini and Hochberg (Benjamini Y and Y, 1995). The scripts for the analysis performed in this study can be accessed at https://github.com/TransBioInfoLab/pathwayMultiomics_manuscript_supplement.

## Results

### Results of the Simulation Study

As discussed in Methods, pathwayMultiomics has two approaches for computing *p-*values, either by approximation using formula or by simulation. Our results showed the estimated parameters *α* and *β* for Beta distribution based on simulation are *α* = 1.85 and *β* = 1.9, which are very similar to the theoretical values of *α* = 2 and *β* = 2 used in the approximation approach. The results in Supplementary Table 1 showed that both the simulation and approximation approaches had Type-I error rates close to 5%. Therefore, we next compared AUCs for the pathwayMultiomics method in the approximation approach with the other four methods.

Among all methods, the pathwayMultiomics method performed best with the highest AUCs across all 20 simulation scenarios (Figure 2, Supplementary Table 2). The second-best performing method is mitch, for which ranking genes by *p-*values performed better than ranking genes by *t*-statistic in most simulation scenarios, except the ones with weak association signals (i.e., effect size = 0.1). The iProFun method also performed well in the simulated pathways that included a high proportion (e.g., 80%) of genes with large association signals (e.g., effect size = 0.5). On the other hand, the sparse mCCA and MFA methods lacked power, probably because these matrix factorization techniques lost information by requiring matched samples or genes across all platforms, and their unsupervised framework also ignored phenotype information. Because sparse mCCA lacked power even in the last simulation scenario with the strongest signal (80% genes in a true positive pathway are treated with an effect size of 0.5), we only included AUC for sparse mCCA in the last simulation scenario.

### Case Study: Analysis of Multi-Omics Datasets in Alzheimer’s Disease

We next applied the two methods that performed best in our simulation study, pathwayMultiomics and mitch, to analyze a collection of real multi-omics datasets in Alzheimer’s disease, which included summary statistics for genetic variants and DNA methylation from two recent large-scale meta-analysis studies (Kunkle et al., 2019; Zhang et al., 2020), as well as a gene expression dataset measured on the prefrontal cortex of brain samples generated by the ROSMAP study (De Jager et al., 2014; De Jager et al., 2018). Note that because we did not have access to raw genotype data included in the meta-analysis, many of the tools that require raw omics data would not be applicable here. In contrast, pathwayMultiomics and mitch can be applied to analyze summary statistics obtained in meta-analyses. For comparison, we also included a third method, the commonly used Venn diagram method, which identifies pathways that are significant in multiple omics data types.

**FIGURE 3:**
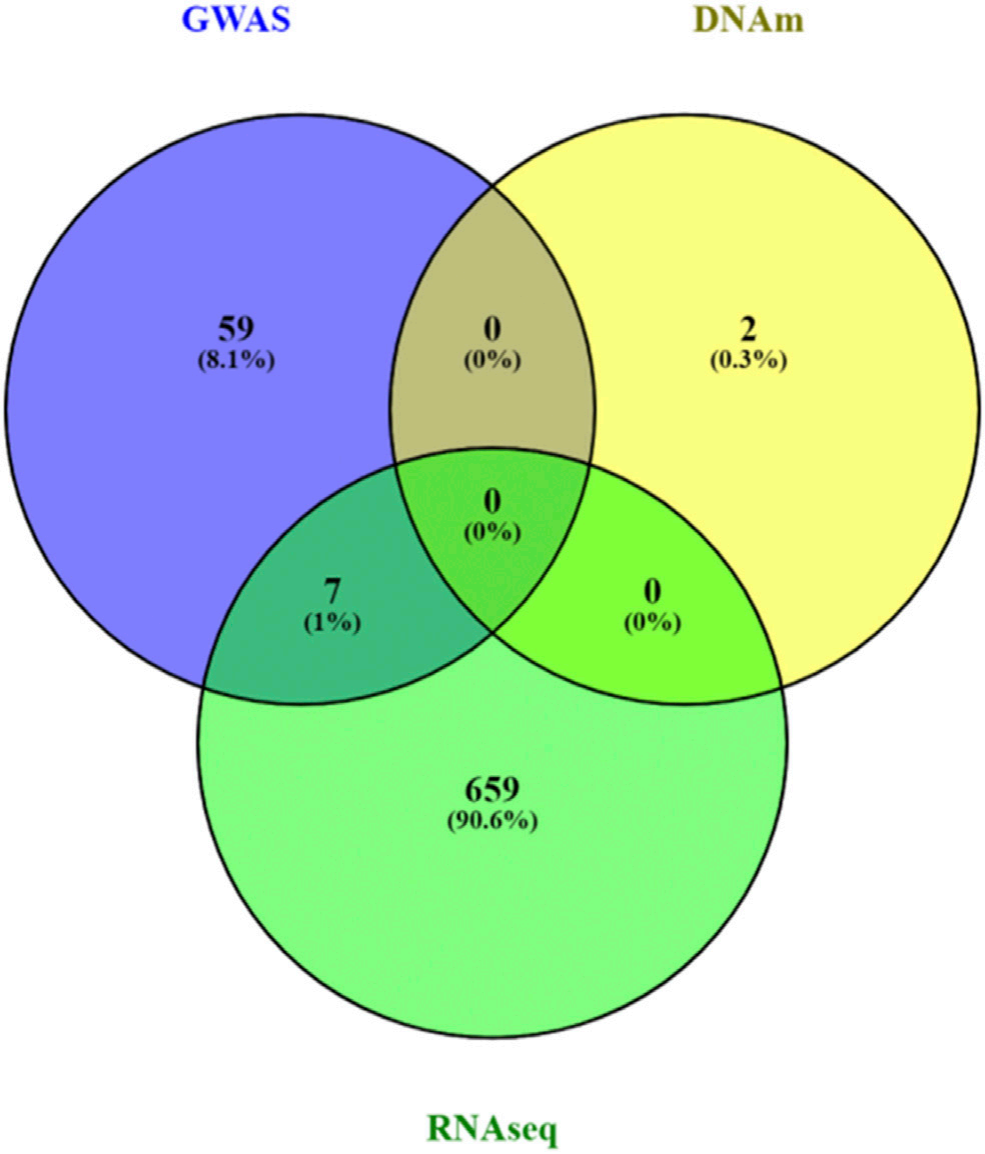
Venn diagram of pathway analyses results for individual omics data types. A total of 666, 2 and 66 significant pathways reached 5% false discovery rate in the analyses of GWAS, DNA methylation (DNAm) and RNASeq data pathway analyses, respectively. Very few pathways (n = 7) were significantly associated with AD in more than one omics data types. The mixed models approach, MissMethyl, and fgsea, which were specifically designed for pathway analyses of genetic variants, DNAm, and gene expression data were used to analyze a total of 2,833 canonical pathways in MsigDB database.

We analyzed 2,833 canonical pathways (C2:CP collection) in MSigDB (Subramanian et al., 2005) that included between 3 and 200 genes. Analyzing each omics data type individually, at a 5% false discovery rate (FDR), we identified 66, 2, and 666 pathways associated with AD in SNP, DNAm, and gene expression data, respectively (Supplementary Table 3–5). There was little agreement between the FDR-significant pathways identified in different omics datasets (Figure 3). A possible reason could be the lack of power in single omics studies for Alzheimer’s disease, which has relatively weaker association signals than other complex diseases such as cancers. Among the top pathways, only seven pathways reached 5% FDR in more than one omics data type. These seven pathways, which reached 5% FDR in both GWAS and RNASeq analysis, are MHC Class II antigen presentation, TCR signaling, factors involved in megakaryocyte development and production, Rig I like receptor signaling pathway, DDX58 IFIH1 mediated induction of interferon alpha-beta, and regulation of toll-like receptor signaling pathway, all of which are involved in inflammatory responses, highlighting the importance of immune processes in AD (Cunningham, 2013; Heneka et al., 2015).

At 5% FDR, pathwayMultiomics identified 74 significant pathways (Supplementary Table 6). Note that for this analysis example, the MiniMax statistics in pathwayMultiomics is the minimum of all maximums in pairs of *p-*values from individual omics, that is min{ max (SNP pathway *p-*value, DNAm pathway *p-*value), max (SNP pathway *p-*value, RNAseq pathway *p-*value), max (DNAm pathway *p-*value, RNAseq pathway *p-*value) }. For these significant pathways, we next examined which two omics data types contributed to the MiniMax statistics. Among the 74 pathways, the significance of the pathwayMultiomics *p-*value (for MiniMax statistic) was driven by pathway *p-*values for DNAm and RNA in the majority of pathways (n = 40, 54%), followed by pathway *p-*values for SNP and RNA (n = 25, 34%), recapitulating the prominent gene regulatory role of DNAm in AD (Klein et al., 2016). In contrast, pathwayMultiomics *p-*values were driven by *p-*values for SNP and DNAm in only 9 (12%) out of the 74 significant pathways, consistent with the relatively independent contributions of genetic variants and DNA methylations in influencing AD susceptibility (Chibnik et al., 2015; Klein et al., 2016). The majority of the top 10 most significant pathways identified by pathwayMultiomics (Table 2) involved signaling pathways activated by the immune system in responses to amyloid-β induced neurotoxicity in AD brains, such as the activation of chemokines (Jorda et al., 2020), toll-like receptors (Landreth and Reed-Geaghan, 2009), T cell receptors (Gate et al., 2020), PDGFR-beta receptors (Liu H. et al., 2018), and CXCR4 receptors (Li and Wang, 2017). Notably, seven out of these top 10 pathways did not reach 5% FDR in more than one type of omics in the analysis of individual omics data types (Figure 3), so these pathways would have been missed by the conventional Venn diagram method.

**TABLE 2:** Top 10 most significant pathways identified by pathwayMultiomics in the analysis of multiomics Alzheimer’s datasets.

		Single omics *p*-values			Single omics FDRs			pathwayMultiomics			
Pathway	Size	SNP	DNAm	RNASeq	SNP	DNAm	RNASeq	MiniMax	*p*-value	FDR	Contributing Omics
PID_PDGFRB_PATHWAY	126	6.99E-01	1.45E-04	1.67E-04	9.99E-01	1.37E-01	3.30E-03	1.67E-04	8.33E-08	2.36E-04	DNAm, RNA
WP_CHEMOKINE_SIGNALING_PATHWAY	155	8.19E-01	3.17E-04	1.94E-05	9.99E-01	1.39E-01	9.00E-04	3.17E-04	3.02E-07	3.28E-04	DNAm, RNA
KEGG_HEMATOPOIETIC_CELL_LINEAGE	80	3.67E-36	3.40E-04	7.61E-01	3.24E-34	1.39E-01	8.11E-01	3.40E-04	3.48E-07	3.28E-04	SNP, DNAm
PID_TCR_PATHWAY	58	4.48E-04	2.75E-02	4.90E-04	2.04E-02	6.43E-01	6.55E-03	4.90E-04	7.20E-07	5.10E-04	SNP, RNA
WP_REGULATION_OF_TOLLLIKE_RECEPTOR_SIGNALING_PATHWAY	128	3.76E-05	3.32E-02	6.55E-04	2.08E-03	6.68E-01	7.70E-03	6.55E-04	1.29E-06	5.69E-04	SNP, RNA
KEGG_CHEMOKINE_SIGNALING_PATHWAY	172	7.90E-01	6.72E-04	2.98E-04	9.99E-01	1.47E-01	4.70E-03	6.72E-04	1.35E-06	5.69E-04	DNAm, RNA
PID_KIT_PATHWAY	52	2.55E-01	6.84E-04	1.10E-04	9.99E-01	1.47E-01	2.69E-03	6.84E-04	1.40E-06	5.69E-04	DNAm, RNA
WP_KIT_RECEPTOR_SIGNALING_PATHWAY	57	3.05E-02	3.68E-04	1.41E-03	5.95E-01	1.39E-01	1.26E-02	1.41E-03	5.94E-06	2.10E-03	DNAm, RNA
PID_CXCR4_PATHWAY	98	4.87E-04	1.50E-03	7.29E-02	2.19E-02	2.36E-01	1.56E-01	1.50E-03	6.76E-06	2.13E-03	SNP, DNAm
REACTOME_TCR_SIGNALING	112	6.06E-52	2.07E-01	2.16E-03	6.11E-50	9.46E-01	1.68E-02	2.16E-03	1.40E-05	3.98E-03	SNP, RNA

At 5% FDR, mitch identified 237 pathways (Supplementary Table 7). The most significant pathway pointed to systemic lupus erythematosus (SLE), an autoimmune disease in which the immune system attacks the body’s own tissues. A recent meta-analysis found that patients with SLE have a significantly higher risk for cognitive impairment (Zhao et al., 2018). Other top pathways (Table 3) highlighted key biological processes regulated by proteins previously shown to be important in AD, such as PRC2 (Zhang et al., 2020), which regulates neuronal lineage specification, proliferation, and differentiation (Liu P.-P. et al., 2018); PKN1, which was shown to have a neuroprotective role (Thauerer et al., 2014); and histone deacetylases (HDACS), which maintains the histone acetylation homeostasis and play important roles in the process of neuronal differentiation, neurite outgrowth and neuroprotection (Shukla and Tekwani, 2020).

**TABLE 3:** Top 10 most significant pathways identified by the mitch method in the analysis of Alzheimer’s disease multi-omics datasets.

Pathway	Size	*p-*value	FDR
KEGG_SYSTEMIC_LUPUS_ERYTHEMATOSUS	128	7.49E-19	2.11E-15
REACTOME_SIRT1_NEGATIVELY_REGULATES_RRNA_EXPRESSION	65	3.16E-15	4.46E-12
REACTOME_DNA_METHYLATION	62	1.21E-13	1.14E-10
REACTOME_ACTIVATED_PKN1_STIMULATES_TRANSCRIPTION_OF_AR_ANDROGEN_RECEPTOR_REGULATED_GENES_KLK2_AND_KLK3	64	2.40E-13	1.69E-10
REACTOME_HDACS_DEACETYLATE_HISTONES	91	6.03E-13	3.40E-10
REACTOME_CONDENSATION_OF_PROPHASE_CHROMOSOMES	71	5.28E-12	2.48E-09
REACTOME_HDMS_DEMETHYLATE_HISTONES	45	5.17E-11	2.08E-08
REACTOME_FORMATION_OF_THE_CORNIFIED_ENVELOPE	129	4.21E-10	1.48E-07
REACTOME_PRC2_METHYLATES_HISTONES_AND_DNA	70	5.40E-10	1.69E-07
REACTOME_TRANSCRIPTIONAL_REGULATION_OF_GRANULOPOIESIS	88	6.79E-10	1.91E-07

**FIGURE 4:**
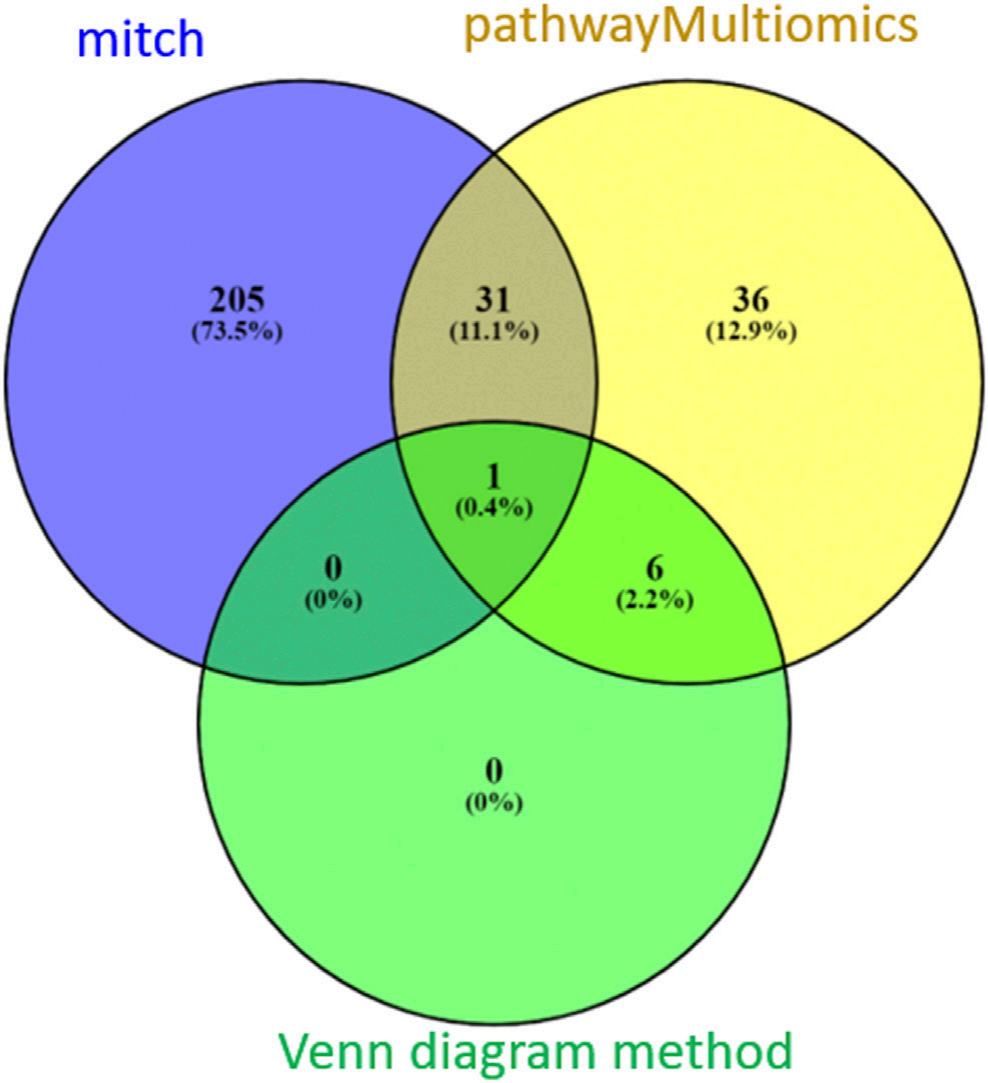
A comparison of FDR significant pathways identified by pathwayMultiomics, mitch, and Venn diagram analyses. At 5% FDR, pathwayMultiomics and mitch identified 74 and 237 pathways, respectively. The Venn diagram method identified 7 pathways with 5% FDR in more than one type of omics data type. There was only modest overlap between the three methods. A total of 32 pathways (11%) were significant in both pathwayMultiomics and mitch methods. PathwayMultiomics identified all the significant pathways using the Venn diagram method. There was no overlap between significant pathways by mitch and Venn diagram, except for one pathway (T cell Receptor pathway), which was identified by all three methods.

Between the three methods (pathwayMultiomics, mitch, and Venn diagram), there was only modest overlap (Figure 4). A total of 32 pathways (11%) reached 5% FDR by both pathwayMultiomics and mitch methods. PathwayMultiomics identified all seven significant pathways that were significant in more than one type of omics data type based on the Venn diagram method. There was no overlap between significant pathways by mitch and Venn diagram method, except for one pathway (T cell Receptor pathway), which was identified by all three methods.

## Discussion

To identify pathways dysregulated in multiple types of omics datasets, we developed the pathwayMultiomics R package. PathwayMultiomics is flexible and only requires pathway *p-*values for individual omics data types as input, thus making it possible to take advantage of pathway analysis tools that are specially designed for each omics data type. In addition, pathwayMultiomics is computationally efficient, does not require matched samples from multi-omics data, and is applicable in situations when raw omics data are not available, such as when aggregating summary statistics from meta-analyses related to the same disease. PathwayMultiomics is also informative; the individual omics data type that contributed to pathwayMultiomics significance can be used to distinguish pathways with potentially different underlying regulatory mechanisms, such as the pathways for which gene expressions are regulated by DNA methylation versus pathways for which gene expressions are mainly regulated by genetic variants.

We performed a comprehensive simulation study to assess the statistical properties of our method. To emulate correlation patterns in real omics datasets, we generated simulation datasets using real TCGA multi-omics datasets as input. We showed that pathwayMultiomics significantly outperforms currently available multi-omics methods with improved power and well-controlled false-positive rates. A challenge with analyzing multi-omics datasets is that many of the samples with data recorded for one molecular type did not have matching data from other data types. Therefore, methods that require matched samples across all data types (e.g., mSCCA) would only analyze a subset of the samples, which would result in reduced statistical power. Also, often only a subset of genes is measured by multiple omics platforms. Therefore, methods that require the same set of genes measured on all omics data types (e.g., MFA) may also exclude important biological signals, leading to reduced power. Finally, unsupervised methods (e.g., NMF, sCCA, and iProFun) might also lose power because they do not leverage information in the phenotypes. In contrast, pathwayMultiomics gains power by leveraging information in all samples (including the un-matched samples), and all features (e.g., genes) mapped to the pathways, as well as phenotype information along with multi-omics data.

To further assess the performance of pathwayMultiomics on real datasets, we also compared it with two alternative approaches using the Venn diagram and mitch. When multiple types of omics data are available, a commonly used strategy is to test for marginal associations between each type of omics data with phenotype first, and then use Venn diagram to intersect significant pathways or genes that overlap in different omics data types. Although a good visualization tool, Venn diagrams do not provide prioritization or any statistical assessment for pathways. In addition, it might be overly stringent because when several types of omics data are considered, often few (if any) pathways pass the threshold of statistical significance in all omics data types. In contrast, pathwayMultiomics provides prioritization and statistical assessment for pathways with moderate to strong association signals in multiple omics data types. In our analysis of multi-omics AD datasets, at 5% FDR, pathwayMultiomics identified 67 pathways in addition to the seven FDR-significant pathways in more than one type of omics data as identified by the Venn diagram method. The discrepancy in multi-omics analysis results by pathwayMultiomics and mitch is not unexpected. In addition to the differences in underlying algorithms, an important reason might also be the different hypotheses these methods test. While mitch tests the competitive null hypothesis that the genes in a pathway show the same magnitude of associations with the disease phenotype compared with genes in the rest of the genome, pathwayMultiomics tests the self-contained null hypothesis that the genes in a pathway are not associated with the disease phenotype (Tian et al., 2005). Therefore, mitch and pathwayMultiomics analysis complement each other in the analysis of multi-omics datasets. PathwayMultiomics is available as an R package and can be accessed at https://github.com/TransBioInfoLab/pathwayMultiomics.

## Conclusions

In summary, we have presented the pathwayMultiomics method, which can be used to analyze multi-omics data with any type of outcome variables (e.g., categorical, continuous, or survival phenotypes). We have shown that pathwayMultiomics significantly outperforms currently available multi-omics methods with improved power and well-controlled false-positive rates. In addition, we also analyzed multi-omics datasets in Alzheimer’s disease to show that pathwayMultiomics was able to recover known biology, as well as nominate novel biologically meaningful pathways. We expect pathwayMultiomics to be a useful tool for integrative analysis of multiple types of omics data.

## Data Availability

The TCGA cancer datasets can be accessed from the LinkedOmics repository http://linkedomics.org/login.php, the Alzheimer’s GWAS summary statistics can be accessed from https://www.niagads.org/igap-rv-summary-stats-kunkle-p-value-data (file “Kunkle_et al._Stage1_results.txt”), the ROSMAP RNASeq dataset can be accessed from AMP-AD (accession: syn3388564). The pathwayMultiomics software can be accessed at https://github.com/TransBioInfoLab/pathwayMultiomics The scripts for the analysis performed in this study can be accessed at https://github.com/TransBioInfoLab/pathwayMultiomics_manuscript_supplement The benchmark dataset used in the simulation study is available at https://zenodo.org/record/5683002#.YZF5SGDMKUk.
